# Leukoaraiosis and Gray Matter Volume Alteration in Older Adults: The PROOF Study

**DOI:** 10.3389/fnins.2021.747569

**Published:** 2022-01-13

**Authors:** Sébastien Celle, Claire Boutet, Cédric Annweiler, Romain Ceresetti, Vincent Pichot, Jean-Claude Barthélémy, Frédéric Roche

**Affiliations:** ^1^Clinical Physiology, Visas Center, University Hospital, Saint-Etienne, France; ^2^INSERM, U1059, SAINBIOSE, DVH, Saint-Étienne, France; ^3^Department of Radiology, University Hospital, Saint Etienne, France; ^4^EA7423 TAPE, UJM, Saint-Étienne, France; ^5^Department of Geriatric Medicine and Memory Clinic, Research Center on Autonomy and Longevity, University Hospital, Angers, France; ^6^UPRES EA4638, University of Angers, Angers, France

**Keywords:** leukoaraiosis, aging, voxel based morphometry, cognition, vascular risk factors

## Abstract

**Background and Purpose:** Leukoaraiosis, also called white matter hyperintensities (WMH), is frequently encountered in the brain of older adults. During aging, gray matter structure is also highly affected. WMH or gray matter defects are commonly associated with a higher prevalence of mild cognitive impairment. However, little is known about the relationship between WMH and gray matter. Our aim was thus to explore the relationship between leukoaraiosis severity and gray matter volume in a cohort of healthy older adults.

**Methods:** Leukoaraiosis was rated in participants from the PROOF cohort using the Fazekas scale. Voxel-based morphometry was performed on brain scans to examine the potential link between WMH and changes of local brain volume. A neuropsychological evaluation including attentional, executive, and memory tests was also performed to explore cognition.

**Results:** Out of 315 75-year-old subjects, 228 had punctuate foci of leukoaraiosis and 62 had begun the confluence of foci. Leukoaraiosis was associated with a decrease of gray matter in the middle temporal gyrus, in the right medial frontal gyrus, and in the left parahippocampal gyrus. It was also associated with decreased performances in memory recall, executive functioning, and depression.

**Conclusion:** In a population of healthy older adults, leukoaraiosis was associated with gray matter defects and reduced cognitive performance. Controlling vascular risk factors and detecting early cerebrovascular disease may prevent, at least in part, dementia onset and progression.

## Introduction

Leukoaraiosis is a rarefaction of white matter found on magnetic resonance imaging (MRI) as white matter hyperintensities (WMH) (Hachinski et al., 1987). WMH are commonly observed on brain MRIs in older adults, thus leading some authors to speak of age-related white matter changes.

Although they are considered as silent manifestations of aging, WMH do not only correlate with age but they are also associated with numerous cardiovascular risk factors including hypertension, diabetes mellitus (Scharf et al., 2019; Grosu et al., 2021), and obstructive sleep apnea (Ho et al., 2018). We already reported that, in older untreated prehypertensive patients, increased ambulatory blood pressure (BP) was significantly associated with higher occurrence of leukoaraiosis (Avet et al., 2014), an alteration found with a relative low mean load value of 24-h systolic BP. Such frailty of the peripheral vascular cerebral tree in older adults challenges the general practitioners to apply a more important use of antihypertensive medication in older adults, optimal in fact, and consider the other risk factors of obstructive sleep apnea and diabetes as well. It is noticeable that these three conditions share hypertension as a common risk factor.

Furthermore, leukoaraiosis appears to be deleterious for cognitive functions, resulting in lower hippocampal volume (Triantafyllou et al., 2020) and/or higher prevalence of mild cognitive impairment, Alzheimer’s disease, and dementia (Cai et al., 2015; Alber et al., 2019). The pathophysiological consequences of such alterations in deep brain white matter are still debated. It is proposed that axonal alterations linked to microvascular periventricular occlusion may in turn affect the integrity of neuronal function, with acceleration of cell death in several critical cortical areas. In 2018, Al-Janabi et al. (2018) observed that WMH were one of the major factors explaining global cerebral atrophy. Furthermore, Lambert et al. (2015, 2016) observed significant gray matter (GM) changes linked to WMH. According to Raji et al. (2012), the increase in white matter lesions is related to a decrease in GM volume as well as a decrease in cognition. To our knowledge, the link between WMH, a full exhaustive exploration of cognitive decline, and GM decrease has never been studied in the healthy elderly population.

Hence, our aim in the present study was to refine this three-party relationship between leukoaraiosis severity and GM volume decrease in a cohort representative of the general population aged 75 years and over.

## Materials and Methods

### Population

The PROgnostic indicator OF cardiovascular and cerebrovascular events (PROOF) study consists of 1,011 healthy older adults homogeneous in age, 65 years, when recruited for inclusion in the city of Saint-Étienne, France (Barthélémy et al., 2007). At inclusion, in 2001, all participants were at low risk of cardiac or cerebrovascular events (2001 timepoint).

In 2009–2011, participants were invited to pass the fourth examination of the study comprising ambulatory BP monitoring and MRI of the brain. More than a half of the 1,011 subjects agreed (2010 time point).

The PROOF study was approved by an Ethics Review Board (CCPRB Rhône-Alpes Loire), and all participants signed a written informed consent for all clinical research procedures.

### Ambulatory Blood Pressure Monitoring

Ambulatory blood pressure was monitored on 24 h using a non-invasive auscultatory method (Diasys Integra, Novacor, Rueil-Malmaison, France) at both time points (Avet et al., 2014). Automatic measurements began in the early morning on a weekday and were taken from the non-dominant arm every 15 min during daytime and every 30 min during the night. During the recording, participants were required not to change their habits, including daily activities and sleeping habits.

Clinical systolic and diastolic blood pressures (SBP and DBP, respectively) were also measured and defined as the mean of two consecutive BP measurements obtained with a mercury sphygmomanometer after the subject had been lying down for 15 min.

Average values of 24-h SBP and DBP, and awake and sleep SBP and DBP, were calculated from ambulatory blood pressure monitoring (ABPM). Systolic and diastolic Dip were calculated according to Dip = (awake BP − sleep BP/awake BP); pulse pressure (PP) was defined as PP = 24-h SBP − 24-h DBP; and mean arterial BP (MAP) was obtained from MAP = (24-h SBP + [2 × 24-h DBP])/3).

### Brain Magnetic Resonance Imaging

MRI was performed at a second time point with a 1.5-T scanner (Magnetom Avento, Siemens Healthcare, Erlangen, Germany), which had a 12-channel head coil. The acquisition protocol included a 3D T1 magnetization prepared rapid gradient echo (MP-RAGE) with a resolution of 1 × 1 × 1 mm^3^, repetition time (TR) of 2,060 ms, echo time (TE) of 3.23 ms, inversion time (TI) of 1,100 ms, and acquisition matrix of 256 × 256 and included 176 slices. The protocol also included a T2-weighted, 2D turbo spin echo with a TR of 6,270 ms, TE of 109 ms, acquisition matrix of 448 × 360, and 24 slices which were 5 mm thick. A 2D fluid-attenuated inversion recovery (FLAIR) was also acquired with a TR of 5,000 ms, TE of 350 ms, TI of 1,800 ms, acquisition matrix of 256 × 192, and 52 3-mm-thick slices.

Visual examination of the MRIs lead to the exclusion of scans considered as structurally invalid for a voxel-based morphometry (VBM) study (meningiomas, major lacunas, cysts, sequels of stroke).

### Assessment of White Matter Lesions

The degree of white matter lesion severity was rated using the semiquantitative visual age-related white matter changes scale (ARWMC) devised by Fazekas et al. (2002) applied to the T2-weighted FLAIR images. ARWMC was graded on a four-point scale of increasing severity: 0, normal; 1, punctuate foci; 2, beginning confluence of foci; and 3, large confluent areas. The reliability of the Fazekas scale is high with an intraoperator correlation coefficient of 0.85 (Leaper et al., 2001), and MR images were scored by a single neuroradiologist observer (CB) who was blinded to the participants’ clinical information, including age, gender, prior imaging findings, and cardiovascular disease risk factors.

### Cognitive Evaluation

Cognitive evaluation was performed using a battery of psychometric tests (Saint Martin et al., 2012). Global intellectual performance was evaluated using the Mini Mental State Examination. Cognitive complaint was investigated with a visual analog scale. We then explored more precisely three cognitive domains: memory, attention and information processing, and executive functioning.

Memory was assessed using the Benton Visual Retention Test (Form C) and the Free and Cued Selective Reminding Test (FCSRT) from Grober & Buschke. Attention and information processing were assessed using the Trail-Making Test Part A (TMTA), the Stroop Color–Word Test, and the coding subtest of the Wechsler Adult Intelligence Scale III (WAIS III). Executive functioning was tested using the Trail-Making Test Part B (TMTB), the Stroop Color–Word Test, an alphabetic fluency test using the letter P, a category fluency test using animal names, and the similarities subtest of the WAIS III.

We also assessed anxiety and depression using the Pichot questionnaire and the French version of the Goldberg scale, respectively. Cognitive function was assessed using the short form of the MacNair scale.

### Voxel-Based Morphometry

MRI images were processed according to classical SPM12 protocol using default settings. All images were first segmented using SPM12 segmentation, formerly known as NewSegment method. Gray matter, white matter, and cerebrospinal partitions were visually checked for mis-segmentation. Preregistered segmented white and gray matter images were registered using the DARTEL algorithm after the creation of a template based on all scans. Registered segmented images were normalized to the Montreal Neurological Institute (MNI) space and were finally smoothed with an 8*8*8-mm^3^ kernel. Mis-registration was visually checked, and a CAT12 check sample homogeneity script was used to focus on the most different images. No subject was excluded after the visual inspections.

### Statistical Analysis

For VBM analysis, we performed a multiple linear regression with SPM12: leukoaraiosis was the covariate of interest whereas sex, presence of antihypertensive medications, sociocultural level, and total intracranial volume were introduced in the model as covariates. The statistical threshold was set at *p* < 0.05 at the family-wise error (FWE)-corrected voxel level with an extent threshold of 250 voxels to have the most significant clusters (*p* < 0.001 at cluster level).

One-way ANOVA was used to explore the differences of cognitive tests between each level of leukoaraiosis. The Scheffe *post hoc* test was used to identify which of the three groups were statistically different.

## Results

### Characteristics of the Population

**FIGURE 1:**
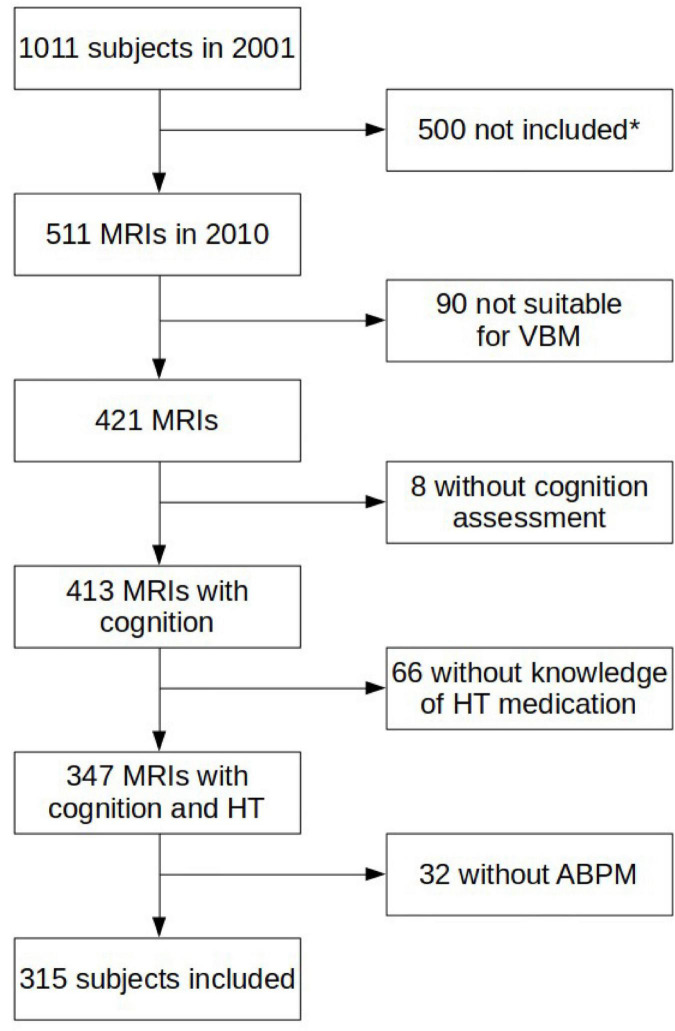
Detailed description of the inclusion/exclusion. MRI: magnetic resonance imaging, HT: hypertension, ABPM: ambulatory blood pressure monitoring. *Missing subjects were majorly dead but were also lost from view or withdrew from the study. Others were not eligible for MRI (claustrophobia, pacemaker) or refused MRI.

From the 1,011 subjects included in 2001, five hundred did not have an MRI recording and 90 MRIs were considered as invalid. Eight more subjects were excluded because they did not have the cognition assessment. We had no information about medications in 66 more subjects. Furthermore, 32 refused the ABP monitoring or had invalid data. Three hundred and fifteen participants were finally included in the present analysis (Figure 1).

At the time of the MRI recording, participants were 75.3 ± 0.8 years old and slightly overweight with a BMI of 25.1 ± 3.6. Detailed characteristics of the population are given in Table 1.

**TABLE 1:** Characteristics of the population.

	Mean ± SD	[Min–Max]
Age (years)	75.3 ± 0.8	[72.3–77.8]
BMI (kg/m^2^)	25.1 ± 3.6	[15.1–35.8]
Waist circumference (cm)	86.9 ± 10.8	[62–117]
Fasting blood glucose (g/L)	1.0 ± 0.2	[0.7–2.4]
Total cholesterol (g/L)	2.3 ± 0.4	[1.2–3.5]
HDL cholesterol (g/L)	0.7 ± 0.8	[0.3–1.3]
LDL cholesterol (g/L)	1.4 ± 0.3	[0.4–2.4]
LDL/HDL cholesterol	2.1 ± 0.7	[0.3–4.2]
Triglycerides (g/L)	1.1 ± 0.5	[0.4–4.0]
SBP (mmHg)	133.2 ± 19.4	[76–189]
DBP (mmHg)	85.6 ± 15.2	[44–149]
24-h SBP (mmHg)	117.2 ± 13.0	[79–159]
24-h DBP (mmHg)	71.9 ± 7.4	[51–98]
Awake SBP (mmHg)	122.0 ± 13.6	[84–162]
Awake DBP (mmHg)	75.2 ± 8.3	[53–117]
Sleep SBP (mmHg)	106.0 ± 13.9	[69–161]
Sleep DBP (mmHg)	64.7 ± 7.9	[45–87]
Systolic dip (mmHg)	0.1 ± 0.1	[−0.2 –+ 0.3]
Diastolic dip (mmHg)	0.1 ± 0.1	[−0.1 –+ 0.5]
PP (mmHg)	45.3 ± 9.6	[21–78]
MAP (mmHg)	87.0 ± 8.5	[63.0–117.7]

*Age, BMI, waist circumference, SBP, and DBP were available for the 315 subjects. Biological data were available for 288 subjects except LDL, HDL, and LDL/HDL cholesterol (287 subjects). ABPM data were available for 309 subjects except for systolic and diastolic DIP (307 subjects).*

*BMI, body mass index; HDL, high-density lipoprotein; LDL, low-density lipoprotein; SBP, systolic blood pressure; DBP, diastolic blood pressure; PP, pulse pressure; MAP, mean arterial pressure; ABPM, ambulatory blood pressure monitoring. Data are presented as mean ± standard deviation.*

### Leukoaraiosis

Out of the 315 subjects, 25 were rated as normal on the ARWMRC, 228 were rated as having punctuate foci, and 62 were rated as presenting beginning confluence of foci. None had large confluent areas.

### Cognition

Leukoaraiosis was mainly associated with differences in memory recall (3-word MMSE recall, total recall, and delayed free recall of the FCSRT) and in executive functioning (TMT-B, Code of the WAIS III) and also associated with depression (Table 2).

**TABLE 2:** Relationship between cognitive tests and leukoaraiosis level using one-way ANOVA.

Leukoaraiosis level	0	1	2	*p*-value
Visual analog scale	2.5 ± 1.6	2.8 ± 1.7	3.0 ± 1.6	ns
MMSE				
Total score	28.4 ± 2.2	28.5 ± 1.6	27.8 ± 2.0	<0.05
3-word recall	2.5 ± 0.8	2.7 ± 2.6	2.5 ± 0.8	ns
FCSRT				
Immediate recall	15.4 ± 0.9	15.4 ± 1.0	15.1 ± 1.2	ns
Total recall	46.6 ± 1.8	46.2 ± 3.0	44.7 ± 6.6	<0.05
Delayed recall	15.8 ± 0.5	15.7 ± 0.7	15.2 ± 2.3	<0.01
Total free recall	29.2 ± 6.6	29.4 ± 5.8	27.7 ± 7.1	ns
Delayed free recall	11.7 ± 2.4	11.7 ± 2.3	11.1 ± 3.1	ns
Delayed cued recall	4.0 ± 2.4	4.1 ± 2.1	4.2 ± 2.4	ns
Intrusions	0.6 ± 1.0	0.7 ± 1.4	1.3 ± 2.	<0.05
Recognition	16.0 ± 0.2	16.0 ± 0.2	15.8 ± 0.7	<0.001
Semantically related intrusions	0	0.0 ± 0.3	0.1 ± 0.9	ns
Unrelated intrusions	0	0.0 ± 0.1	0.0 ± 0.2	ns
Benton test	13.0 ± 1.3	12.6 ± 1.6	12.5 ± 1.6	ns
WAIS-III code test				
Score	50.9 ± 9.6	53.6 ± 12.6	49.0 ± 12.8	<0.05
Standardized score	11.0 ± 2.1	11.6 ± 2.6	10.6 ± 2.8	<0.05
Copy score	110.0 ± 23.4	114.2 ± 20.5	110.2 ± 23.9	ns
Trail making test				
TMT-A (s)	49.7 ± 15.1	46.9 ± 15.4	49.9 ± 16.6	ns
TMT-A Errors	0.0 ± 0.2	0.1 ± 0.8	0.1 ± 0.2	ns
TMT-B (s)	102.3 ± 33.2	98.7 ± 35.8	114.9 ± 58.7	<0.05
TMT-B Errors	0.2 ± 0.4	0.5 ± 1.0	0.7 ± 1.2	ns
TMT-B–TMT-A (s)	52.6 ± 25.6	51.8 ± 30.3	65.0 ± 49.2	<0.05
Stroop				
Word score	89.4 ± 16.9	94.5 ± 13.0	93.0 ± 11.7	ns
Color score	65.9 ± 11.4	66.7 ± 10.7	64.6 ± 8.9	ns
Color–word score	32.3 ± 8.2	32.4 ± 7.9	31.3 ± 9.1	ns
Stroop Interference	3.2 ± 6.6	2.1 ± 6.7	2.1 ± 8.0	ns
Standardized word score	103.4 ± 16.9	108.6 ± 13.0	107.1 ± 11.7	ns
Word z-score	−0.1 ± 0.4	−0.1 ± 0.7	−0.1 ± 0.5	ns
Standardized color score	75.9 ± 11.4	77.6 ± 10.7	75.6 ± 8.9	ns
Color z-score	−0.6 ± 0.7	−0.3 ± 0.7	−0.3 ± 0.7	ns
Standardized color–word score	47.2 ± 8.3	47.3 ± 7.9	46.3 ± 9.0	ns
Color–word z-score	−0.2 ± 0.6	0.1 ± 0.8	−0.4 ± 1.0	ns
Verbal fluency				
Semantic	30.2 ± 8.0	30.4 ± 7.3	28.5 ± 7.7	ns
Semantic false answer	0.8 ± 0.9	0.6 ± 1.0	0.5 ± 0.8	ns
Semantic Z-score	0.1 ± 0.9	0.1 ± 0.9	−0.2 ± 1.0	ns
Phonemic	21.0 ± 8.3	20.4 ± 6.3	19.0 ± 6.4	ns
Phonemic false answer	0.6 ± 0.8	0.6 ± 0.9	0.4 ± 0.8	ns
Phonemic Z-score	0.1 ± 1.0	−0.1 ± 0.9	−0.2 ± 0.7	ns
WAIS-III similarities test				
Score	19.5 ± 5.4	17.7 ± 5.6	17.1 ± 5.8	ns
Standardized score	12.3 ± 2.4	11.4 ± 2.5	11.2 ± 2.8	ns
Z-score	0.8 ± 0.8	0.5 ± 0.8	0.4 ± 0.9	ns
Cognitive function	25.6 ± 8.7	26.8 ± 11.2	29.3 ± 13.9	ns
Anxiety	2.9 ± 2.6	3.1 ± 2.8	3.0 ± 2.6	ns
Depression	2.0 ± 1.7	2.3 ± 2.4	3.5 ± 3.7	<0.001

*MMSE, Mini-Mental State Examination; TMT, trail making test; FCRST, Free and Cued Selective Reminding Test. Data are presented as mean ± standard deviation.*

*Post hoc* analyses revealed that cognition was different between level 2 of the leukoaraiosis scale (beginning confluence) and level 1 (punctuate foci) but not between normal WM and level 1.

### Voxel-Based Morphometry

**FIGURE 2:**
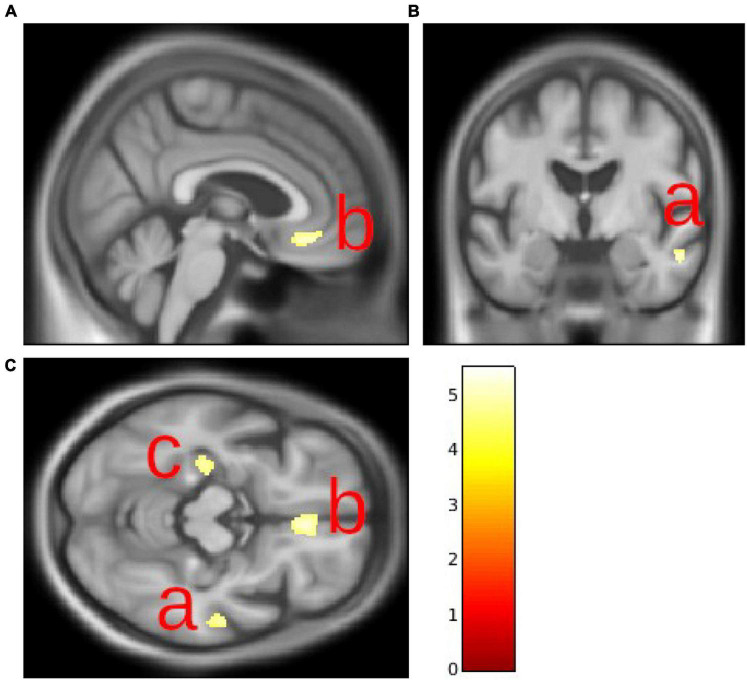
Gray matter volume decrease in the right middle temporal gyrus **(A)**, right medial frontal gyrus **(B)**, and left parahippocampal gyrus **(C)**.

Leukoaraiosis severity at time point 4 was inversely correlated with gray matter volume in the right middle temporal gyrus (Brodmann area 21), in the right medial frontal gyrus (Brodmann area 11), and in the left parahippocampal gyrus (Table 3 and Figure 2). Relationships were statistically significant at the cluster level as well as in the corrected voxel level.

**TABLE 3:** Gray matter decrease associated with an increase in leukoaraiosis score (R: right, L: left, pclust: *p*-value at the cluster level with a family-wise error (FWE) correction, pvox: *p*-value at the peak voxel with a FWE correction).

Side	Brain region	X	Y	Z	Cluster size	pclust	pvox
R	Middle temporal gyrus	54	−8	−22	282	0.001	<0.001
R	Medial frontal gyrus	3	33	−14	337	0.001	0.002
L	Parahippocampal gyrus	−32	−24	−15	265	0.001	0.007

We did not observe any gray matter increase associated with an increase in leukoaraiosis.

## Discussion

The present study confirmed that leukoaraiosis in older adults is associated with a decrease in GM volume in several cortical areas including the right middle temporal gyrus, the right medial frontal gyrus, and the left parahippocampal gyrus. Such decreases remained highly significant after usual corrections for confounders. Moreover, we noticed that higher leukoaraiosis was associated with a decrease in cognitive functions, mainly in memory (total recall) and executive functions (Code of the WAIS III, TMTB).

In 2001, the Leukoaraiosis And DISability (LADIS) study was built to assess the role of white matter changes and their progression on the onset of disability in older adults (The LADIS Study Group, 2011). In 639 non-healthy but functionally autonomous 74-year-old patients, the group demonstrated that WMH were associated with worse score on MMSE (van der Flier, 2005) and worse cognitive performance (Verdelho et al., 2007). However, these subjects had various complaints and disturbances (mild cognitive impairment, motor disturbances, mood disturbances, minor cerebrovascular events, or other neurological problems). Later, the association between WMH and cognitive impairment and/or dementia was confirmed in older adults (94 years old) (Legdeur et al., 2020). A meta-analysis in more than 14,000 participants for WMH showed a link between WMH and dementia/Alzheimer’s disease (AD) (Debette et al., 2019). In 2004, Verdelho et al. (2007) observed an association between WMH and Buschke total recall. We confirmed this association in a healthy 75-year-old population comprising adults without any major cognitive impairment or dementia or cognitive complaint (Capizzano, 2004). The differences observed here are subtle, but they remain statistically significant. Of note, none of the subjects had large confluent areas, as in the publication of Verdelho et al. (2007), while we still noted these significant cognitive variations.

Interestingly, Kim et al. (2020) did not observe any relationship between WMH and cognition on subcortical vascular mild cognitive impairment patients. However, they demonstrated a more rapid cortical thinning in frontal and temporal areas associated with a 3-year WMH progression. Cortical thinning in frontal and temporal areas associated with white matter grade was reported by Raji et al. (2012) as well as a thinning in inferior parietal and parahippocampal areas. For Dickie et al. (2016) WMH were also associated with a cortical thinning within and around the Sylvian fissure. In 2005, van der Flier (2005) from the LADIS study group showed that those with medial temporal atrophy and severe WMH had a fourfold increase in the frequency in mild cognitive deficits. In a meta-analysis by Wang et al. (2021) WMH were also associated with medial temporal lobe atrophy in post-stroke patients. Li et al. (2011) also observed frontal and temporal lobe GM decreases using VBM analysis, as well as decreases in the thalamus and parietal lobe. Several studies reported a link between WMH and hippocampal atrophy in mild cognitive impairment patients (Fiford et al., 2017; Wong et al., 2021). In our study, we also observed a GM volume decrease in similar areas (middle temporal gyrus, medial frontal gyrus) as well as in the parahippocampus. Using linear mixed effect models, van Leijsen and her team explained memory decline in older adults by an interaction between WMH and hippocampus volume (van Leijsen et al., 2019). WMH and hippocampus volume were also both markers of the conversion from normal cognition to mild cognitive impairment in Framingham offsprings (Bangen et al., 2018).

During the past decades, WMH have been associated with the two most common forms of dementia, vascular dementia (VD) and AD. The first form of dementia, VD, is caused by defects in blood supply to the brain which generates lacunae and WMH. The second form of dementia, AD, is a multifactorial neurodegenerative disease whose main risk factors are age, genetic, head injury, and other factors such as high cholesterol or high BP. There is a link between AD and/or dementia and WMH, even after the concomitant effect of age on both pathologies had been dismissed (Sarabia-Cobo et al., 2014; Kao et al., 2019). White matter alterations appear years before the estimated symptomatic onset of the disease in autosomal dominant AD (Lee et al., 2016; Araque Caballero et al., 2018). Rojas et al. (2018) discovered a higher prevalence of WMH in homozygous APOE-ε4 allele carriers aged 45–75 years. In non-genetic forms of AD, this link has been less documented and WMH are only associated with severity of AD. Temporal areas and parahippocampus/hippocampus are the first regions affected in AD. It has been hypothesized that the presence of high WMH and the decrease of gray matter in key brain areas may be viewed as prodromal signs of AD as suggested in the literature (Wolf et al., 2000; Vermeer et al., 2003; Prins et al., 2004). It is of importance to note that our population is highly selected: among the 1,011 healthy participants included in 2001, the 315 participants remaining in this sub-study are probably the healthiest part of the population. Thus, they have shown few WMH and they mainly do not have cognitive impairment. None of these participants suffers from dementia, and we do not yet know whether those with poor cognitive results will convert to dementia or not. Future time points of our study should answer this specific question.

WMH are associated with hypertension, dyslipidemia, or diabetes, suggesting a vascular origin of leukoaraiosis in which hypertension seems to play a major key role (Moroni et al., 2016). As a manifestation of cerebral small vessel disease, leukoaraiosis seems to develop gradually and be the “tip of the iceberg” according to de Groot et al. (2013). Conventional MRI techniques may be not sensible enough to detect the first brain disorders: Altamura et al. (2016) observed on MRIs that even in patients with normal-appearing white matter, higher regional apparent water diffusion coefficients were associated with microcirculatory impairment and altered cognitive domain. It thus seems that WMH can originate from cerebral blood flow decrease (Bernbaum et al., 2015). In a previous study, we observed a linear relationship between leukoaraiosis and BP in untreated for hypertension patients, but not in patients treated for hypertension (Avet et al., 2014), which can suggest that controlling hypertension could lower WMH burden.

However, in our study we only assessed WMH using the Fazekas scale, one among multiple ways to define WMH (Frey et al., 2019). Measurements of WMH volumes or local variations of WMH may bring sharper results. Moreover, we were unable to evaluate the status of our population regarding microbleeds.

As a conclusion, WMH are associated with cognitive impairment and brain GM defects in healthy older adults. As WMH find their origin in cerebrovascular impairment, this result strengthens the assumption by Sudo et al. (2015) that controlling vascular risk factors and detecting early cerebrovascular diseases may prevent, at least in part, dementia onset and progression. Further interventional studies implying the strict control of vascular risk factors and their impact on the brain and dementia should confirm this conclusion.

## Data Availability Statement

The generated datasets may be available after a motivated request to the research team. Requests to access the datasets should be directed to FR.

## Ethics Statement

The studies involving human participants were reviewed and approved by the CCPRB Rhône-Alpes Loire. The patients/participants provided their written informed consent to participate in this study.

## Author Contributions

SC and CB contributed to the analysis and interpretation of data. J-CB and FR contributed to the conception and design of the study. SC drafted the manuscript. All authors critically revised the draft.

## Conflict of Interest

The authors declare that the research was conducted in the absence of any commercial or financial relationships that could be construed as a potential conflict of interest.

## Publisher’s Note

All claims expressed in this article are solely those of the authors and do not necessarily represent those of their affiliated organizations, or those of the publisher, the editors and the reviewers. Any product that may be evaluated in this article, or claim that may be made by its manufacturer, is not guaranteed or endorsed by the publisher.
